# Immune checkpoint inhibitor related hypophysitis: diagnostic criteria and recovery patterns

**DOI:** 10.1530/ERC-20-0513

**Published:** 2021-04-23

**Authors:** Ha Nguyen, Komal Shah, Steven G Waguespack, Mimi I Hu, Mouhammed Amir Habra, Maria E Cabanillas, Naifa L Busaidy, Roland Bassett, Shouhao Zhou, Priyanka C Iyer, Garrett Simmons, Diana Kaya, Marie Pitteloud, Sumit K Subudhi, Adi Diab, Ramona Dadu

**Affiliations:** 1 Division of Internal Medicine, Department of Endocrine Neoplasia and Hormonal Disorders, The University of Texas Anderson Cancer Center, Houston, Texas, USA; 2 Division of Diagnostic Imaging, Department of Diagnostic Radiology, The University of Texas Anderson Cancer Center, Houston, Texas, USA; 3 Division of Science, Department of Biostatistics, The University of Texas Anderson Cancer Center, Houston, Texas, USA; 4 Division of Cancer Medicine, Department of Genitourinary Medical Oncology, The University of Texas Anderson Cancer Center, Houston, Texas, USA; 5 Division of Cancer Medicine, Department of Melanoma Medical Oncology, The University of Texas Anderson Cancer Center, Houston, Texas, USA

**Keywords:** immune hypophysitis, checkpoint inhibitors, hypophysitis diagnostic criteria, hypophysitis recovery

## Abstract

Data on the diagnosis, natural course and management of immune checkpoint inhibitor (ICI)-related hypophysitis (irH) are limited. We propose this study to validate the diagnostic criteria, describe characteristics and hormonal recovery and investigate factors associated with the occurrence and recovery of irH. A retrospective study including patients with suspected irH at the University of Texas MD Anderson Cancer Center from 5/2003 to 8/2017 was conducted. IrH was defined as: (1) ACTH or TSH deficiency plus MRI changes or (2) ACTH and TSH deficiencies plus headache/fatigue in the absence of MRI findings. We found that of 83 patients followed for a median of 1.75 years (range 0.6–3), the proposed criteria used at initial evaluation accurately identified 61/62 (98%) irH cases. In the irH group (*n* = 62), the most common presentation was headache (60%), fatigue (66%), central hypothyroidism (94%), central adrenal insufficiency (69%) and MRI changes (77%). Compared with non-ipilimumab (ipi) regimens, ipi has a stronger association with irH occurrence (*P* = 0.004) and a shorter time to irH development (*P* < 0.01). Thyroid, gonadal and adrenal axis recovery occurred in 24, 58 and 0% patients, respectively. High-dose steroids (HDS) or ICI discontinuation was not associated with hormonal recovery. In the non-irH group (*n* = 19), one patient had isolated central hypothyroidism and six had isolated central adrenal insufficiency. All remained on hormone therapy at the last follow-up. We propose a strict definition of irH that identifies the vast majority of patients. HDS and ICI discontinuation is not always beneficial. Long-term follow-up to assess recovery is needed.

## Introduction

The understanding of the immune system’s role in cancer biology has marked a new era of cancer therapies (Hanahan & Weinberg 2011). The breakthrough discovery of immune checkpoints such as cytotoxic T-lymphocyte antigen 4 (CTLA-4) and programmed cell death 1 (PD-1) had led to the development of immune checkpoint inhibitors (ICI) resulting in substantial changes in cancer treatment paradigms. In 2011, the United States Food and Drug Administration (FDA) approved the first ICI, ipilimumab (anti-CTLA-4 antibody). To date, seven ICIs have been approved by the FDA for various cancer types: anti-CTLA-4 mAb (ipilimumab), anti-PD-1 mAbs (pembrolizumab, nivolumab, cemiplimab) and anti-programmed cell death 1 ligand (PD-L1) mAbs (atezolizumab, durvalumab, avelumab) (Vaddepally *et al.* 2020). Several other immune checkpoints have been described (LAG3, TIM3, KIR, VISTA, CD-40, OX-40, CD-137, GITR, etc.) and agonistic or antagonistic antibodies are being studied in clinical trial setting (Marquez-Rodas *et al.* 2015). Despite ICI’s clinical success, its use poses several challenges and limitations, including off-target immune-related adverse events (irAEs). Endocrine side effects are common and well recognized in clinical practice (Boutros *et al.* 2016). Immune-related hypophysitis (irH) with potentially life-long hormone deficiencies represents a unique irAE that occurs mainly in patients treated with anti CTLA-4 mAbs alone or in combination with other ICIs and remains very rare in patients treated with single agent anti PD-1 or PD- L1 mAbs (Corsello *et al.* 2013, Robert *et al.* 2014, Topalian *et al.* 2014, Chang *et al.* 2019). In a systematic review and meta-analysis including data from 38 randomized clinical trials comprising 7551 patients investigating the use of ICIs in the treatment of various cancer types, irH incidence was reported to range from 1.5% to 13.3% in patients treated with CTLA-4 antibodies and 0.3–3% in those with PD-1 inhibitors (Barroso-Sousa *et al.* 2018). Published case series with review by endocrinologists reported incidence from 11% to 13.3% (Faje *et al.* 2014, Albarel *et al.* 2015, Min *et al.* 2015).

It has been more than a decade since the first cases of the irH were described (Blansfield *et al.* 2005). However, to date, there are no uniform diagnostic criteria and limited information exists on its long-term sequela. In many studies, strict diagnostic criteria of irH as well as its recovery were not established, leading to much difficulty in obtaining accurate data on incidence and prevalence as well as solidifying recommendations of treatment and long-term follow-up. In addition, most studies were small size and multi-institutional with inconsistent information and significant variations in diagnostic work up and approach. Our group have previously proposed a diagnostic criteria for irH based on a small cohort study (Pitteloud *et al.* 2015). In the current study, using a single-center large cohort with long-term follow-up data, we sought to validate irH diagnostic criteria, describe the clinical characteristics of irH and analyze factors that can affect irH occurrence and recovery.

## Methods

### Study population

This is a single-center retrospective chart review study conducted at the University of Texas MD Anderson Cancer Center under an Institutional Research Board approved protocol. All patients on ICIs seen by the endocrine team for suspected irH from 5/2003 to 8/2017 were studied. Patients were included for final analysis if they had completed thyroid and adrenal axis evaluation within 2 weeks of clinical presentation. Pituitary or brain MRI obtained within 4 weeks of clinical presentation was considered eligible for review. Patients were excluded if they were receiving steroids or thyroid hormone doses exceeding 1.6 mcg/kg for pre-existing primary hypothyroidism at the time of first hormonal evaluation, had incomplete thyroid or adrenal hormonal work up, metastasis to the sella, prior radiation therapy involving the sella or were critically ill.

### Diagnostic criteria of irH

The following criteria for irH diagnosis were utilized. Criteria 1: central adrenal insufficiency or central hypothyroidism plus MRI findings consistent with irH or criteria 2: both central adrenal insufficiency and hypothyroidism plus symptom of headache or fatigue in the absence of MRI findings/evaluation.

Central adrenal insufficiency was defined as low cortisol (serum cortisol < 5 mcg/dL) and low/inappropriately normal ACTH or abnormal cosyntropin stimulation test (normal result is defined as serum cortisol ≥ 18 mcg/dL before or after cosyntropin injection (either with low dose 1 mcg or high dose 250 mcg) in the absence of exogenous glucocorticoid use. Central hypothyroidism was defined as low free T4 and low/inappropriately normal TSH in the absence of concurrent thyroid hormone use that exceeds 1.6 mcg/kg/day of levothyroxine or an equivalent dose. Central hypogonadism was defined as low levels of sex hormones and low/inappropriately normal gonadotropins in the absence of androgen deprivation therapy or sex hormone replacement. Normal references at our institution are included in Supplementary Table 1 (see section on supplementary materials given at the end of this article). With regards to imaging studies, the brain or pituitary MRI obtained within 4 weeks of clinical presentation was reviewed and compared with prior and follow-up images. MRI was considered positive for irH when at least two of the following findings were identified: gland height (>2 mm change compared to baseline), suprasellar bulge, stalk thickening, heterogenous enhancement and para sellar extension (Simmons *et al.* 2018). IrH was confirmed and independently reviewed by two board-certified endocrinologists (RD and HN). A review by a third board-certified endocrinologist (SGW) was requested for confirmation if there was a discrepancy. Imaging study was independently reviewed by a board-certified (KS) and a board-eligible radiologist (KD) who were blinded to endocrine review results.

### Diagnostic criteria of hormonal recovery

Adrenal axis recovery was defined as either a peak cortisol of ≥ 18 mcg/dL before or after cosyntropin injection (either low dose 1 mcg or high dose 250 mcg) in a cosyntropin stimulation test or 08:00 h cortisol ≥ 12 mcg/dL with ACTH ≥ 5 pg/mL when steroids were appropriately held for testing. Patients with 08:00 h cortisol < 5 mcg/dL and ACTH < 5pg/mL or failed cosyntropin stimulation test were considered as having no recovery. Thyroid axis recovery was defined as normal TSH and free T4 when the patient was taken off thyroid hormone. Patients with persistent central hypothyroidism while off thyroid hormone or with suppressed TSH and normal free T4 while on thyroid hormone at a dose of less than 1.6 mcg/kg were classified as having no recovery. Gonadal recovery is defined as age-appropriate levels of sex hormones when the patient was off sex hormone replacement. No gonadal recovery is defined as when the patient had sex hormone testing consistent with central hypogonadism while off sex hormone. Adrenal, thyroid and gonadal recovery were considered unclear if patients did not meet the recovery or no recovery criteria.

### Outcomes

In this current study, using a single-center large cohort with long-term follow-up data, we sought to validate previously proposed diagnostic criteria, describe characteristics of irH (symptoms, hormonal work up and imaging findings) and analyze factors that can affect irH occurrence and recovery.

### Statistics

Descriptive statistics were used to report patient baseline characteristics, ICI use, cancer types, time to diagnosis, MRI findings, follow-up duration, and pre-existing autoimmune diseases. To study patterns of MRI resolution and hormonal recovery and identify factors that can affect irH occurrence and hormonal recovery, the method of Kaplan and Meier was used. Univariate Cox proportional hazards regression models were fit to model the time to resolution/recovery with the covariates of interest. For each covariate, the hazard ratio and corresponding 95% CI are presented. For some covariates, the number of events in one category was small, and Firth's penalized likelihood method was used to estimate the hazard ratio and CIs. All statistical analyses were performed using R version 3.4.3. All statistical tests used a significance level of 5%. No adjustments for multiple testing were made.

## Results

### Study population

**Figure 1:**
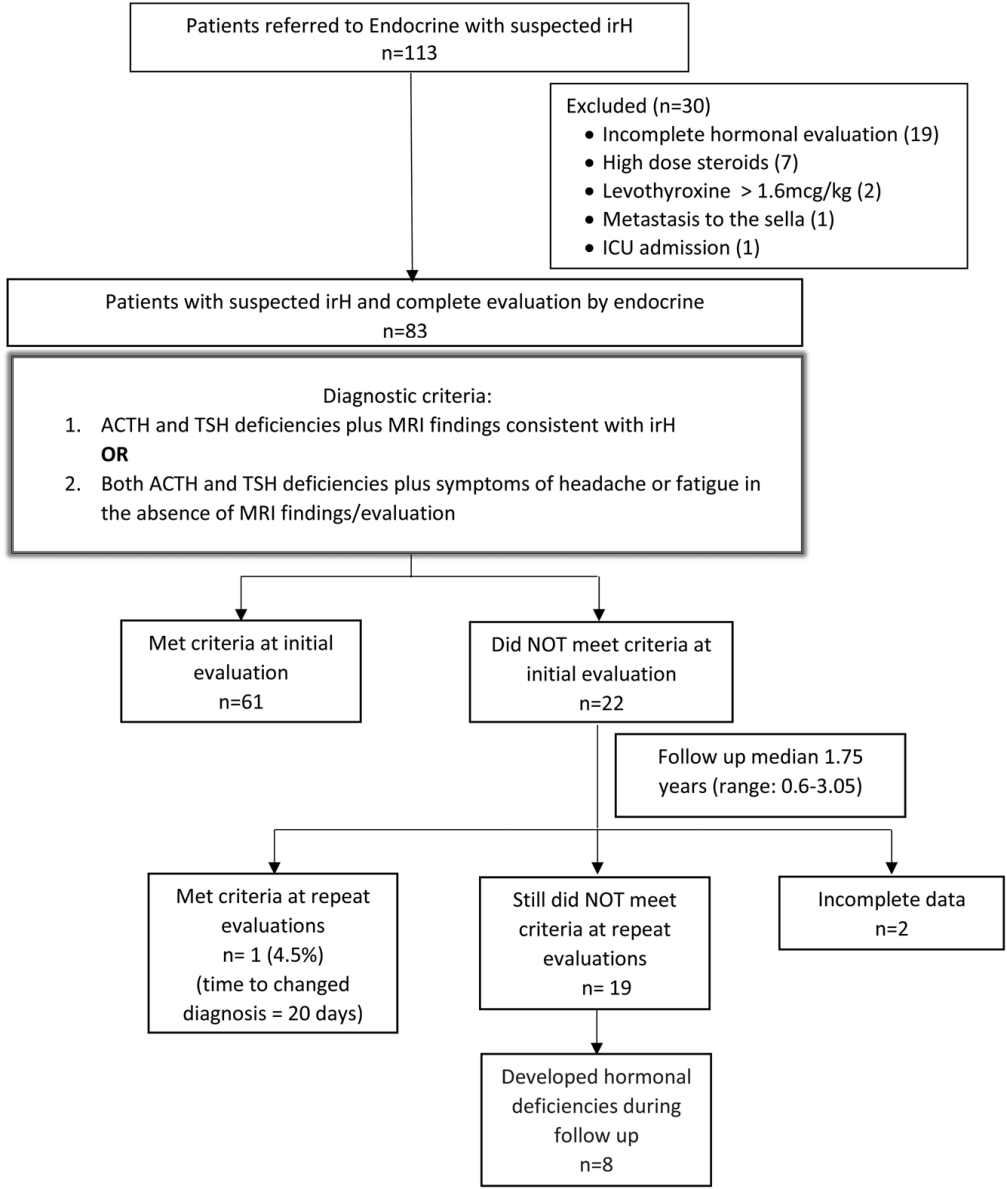
Study population.

Of 113 patients referred to endocrinology for suspected irH, 83 met inclusion criteria. Thirty patients were excluded due to incomplete hormonal evaluation (19), metastasis to the sella (1), critical illness (1), use of high dose steroids for non-irH reasons (7) and levothyroxine dose >1.6 mcg/kg (2). Figure 1 describes the study population. Baseline characteristics of the entire population and subgroups with and without irH are described in Table 1.

**Table 1:** Baseline characteristics of patients with suspected irH who had complete workup by an endocrinologist (*n* = 83) and of patients with confirmed irH (*n* = 62) or not confirmed irH (*n* = 21).

	Total population, (*n* = 83)	IrH confirmed, (*n* = 62)	IrH not confirmed, (*n* = 21)
Age (median, range)	63 (56.4–67)	63.2 (55–67)	61.4 (56.8–69)
Gender, male, *n* (%)	61 (73.5)	48 (77)	13 (62)
Ethnicity, *n* (%)			
Caucasian	74 (89)	56 (90)	18 (86)
Black	6 (7.2)	3 (5)	3 (14)
Hispanic	3 (3.6)	3 (5)	0 (0)
Cancer type, *n* (%)			
Melanoma	54 (65)	42 (68)	9 (43)
Prostate cancer	16 (19.3)	11 (18)	4 (19)
Renal cell carcinoma	8 (9.6)	5 (8)	3 (14)
Immune checkpoint inhibitor, *n* (%)			
Ipilimumab	61 (73.5)	48 (77)	13 (62)
Tremelimumab	2 (2.4)	2 (3)	0 (0)
Nivolumab	6 (7.2)	3 (5)	3 (14)
Nivolumab + Ipilimumab	12 (14.5)	9 (15)	3 (14)
Pembrolizumab	1 (1.2)	0 (0)	1 (5)
Pembrolizumab + Ipilimumab	1 (1.2)	0 (0)	1 (5)

Anti CTLA-4 mAbs (Ipilimumab, Tremelimumab); Anti PD-1 mAbs (Nivolumab, Pembrolizumab); IrH, immune checkpoint inhibitor-related hypophysitis.

### Accuracy of proposed irH diagnostic criteria

Based on the proposed diagnostic criteria, 61/83 (73%) patients had irH at the time of first evaluation, of which 46/61 (75%) patients met the irH diagnosis criteria 1 and 15/61 (25%) patients met criteria 2 (Fig. 1).

Twenty-six of 61 (43%) patients met both criteria. If only clinical data had been used without MRI data, irH diagnosis would have been missed in 20/61 (33%) patients. Twenty-two of 83 (27%) patients did not meet the irH diagnosis criteria at first evaluation. Within this group, during a median follow-up of 1.75 years (0.6–3.05), 1/22 (5%) patients subsequently met the criteria 1 (this patient had brain MRI finding consistent with irH but had no symptoms or hormonal deficiencies and 20 days later developed central hypothyroidism), 2/22 (10%) had incomplete data and 19/22 (85%) still did not meet irH criteria at initial and repeat evaluation. In the studied population, the proposed diagnostic criteria have accurately identified 61/62 (98%) cases with irH at initial evaluation.

Of 19 patients who did not meet irH criteria during the follow-up, one patient (1/19, 5%) had central hypothyroidism and central adrenal insufficiency; however, there were no symptoms or changes in the MRI. One patient (1/19, 5%) had isolated central hypothyroidism without MRI changes. This patient received thyroid hormone replacement and high-dose steroids. At 2 year follow-up, he remained on thyroid hormone. Six patients (6/19, 32%) had isolated central adrenal insufficiency. None of these patients were on steroids or opiods within 6 months prior to the first evaluation of irH. Two patients did not have brain MRI for review while four had MRI images not consistent with irH. Two patients were given high dose steroids for irH treatment and four received a physiologic dose of steroids. All remained on steroids at 2-year follow-up.

### Clinical presentation of irH

**Figure 2:**
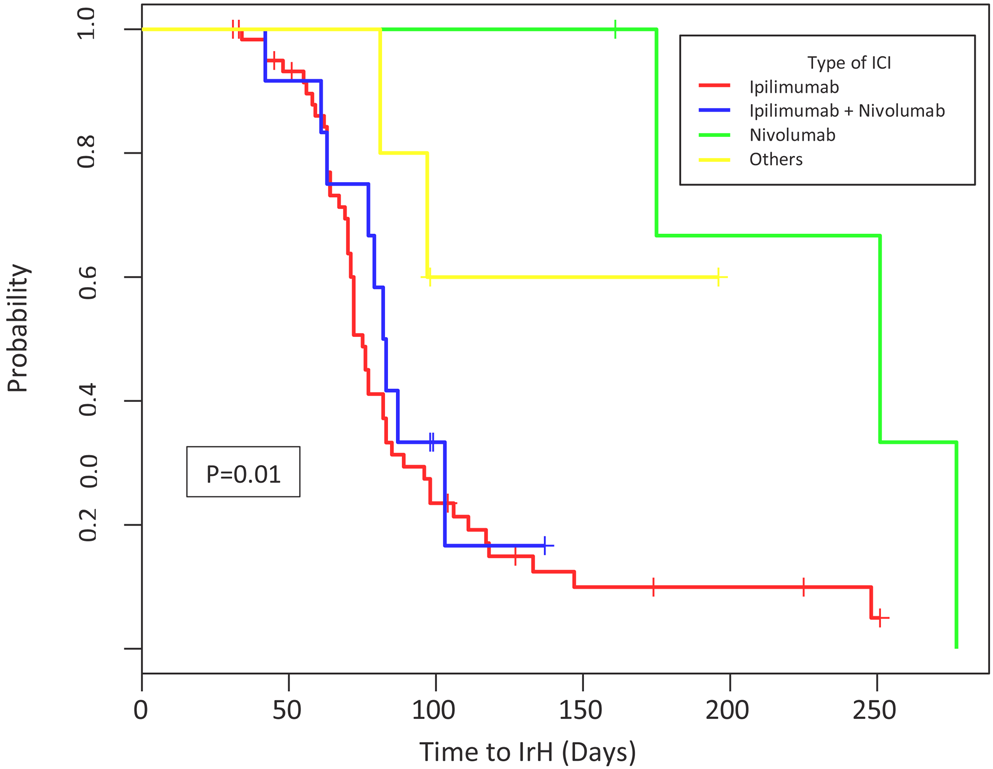
Immune-related hypophysitis development by type of immune checkpoint inhibitors. It presents a Kaplan–Meier plot of time to immune-related hypophysitis (irH) by the type of immune checkpoint inhibitor (ICI). The median time to irH was 10.3 weeks for patients who received ipilimumab, 11.3 weeks for ipilimumab and nivolumab, and 35.8 weeks for nivolumab. There was strong evidence of an association between regimen and time to irH (*P* = 0.01).

Baseline characteristics of irH patients (*n* = 62) are described in Table 1. The median age was 64 (57–67), with 77% male. The majority of patients had melanoma 42/62 (68%), prostate cancer 11/62 (18%), and renal cell carcinoma 5/62 (8%). Other types of cancer were non-small cell lung carcinoma (3%), papillary thyroid carcinoma (2%) and chronic myelomonocytic leukemia (2%). Forty-eight of 62 (77%) patients were on ipilimumab, 2/62 (3%) on tremelimumab, 3/62 (5%) on nivolumab and 9/62 (15%) on a combination of both nivolumab and ipilimumab. Four of 62 (6%) patients had a history of other autoimmune diseases prior to ICI use. Median time from ICI first administration to the diagnosis of irH for the entire population was 11 weeks (9–12.6), 10.3 weeks (9–12) for patients who received ipilimumab, 12.7 weeks (12.1–13.2) for tremelimumab, 11.3 weeks (9–12) for ipilimumab and nivolumab, and 35.8 weeks (30.4–37.7) for nivolumab. There is strong evidence that patients who received ipilimumab (either as a single agent or in combination with nivolumab) developed irH more quickly than patients who did not (*P* < 0.01) (Fig. 2). Median time to irH between the group on ipilimumab alone vs combination of ipilimumab and nivolumab had no statistically significant difference (*P* = 0.6).

Clinical presentation, MRI findings and pituitary hormonal deficiencies are presented in Table 2. Headache and fatigue occurred in 60% and 66% of patients, respectively. No patients had visual changes and diabetes insipidus. The most common hormone deficiency was central hypothyroidism (58/62, 94%), followed by central adrenal insufficiency (43/62, 69%) and hypogonadism (29/57, 50%). Involvement of all three pituitary axes occurs in 18/57 (32%) patients. Low prolactin and IGF-1 levels were present in 27/43 (63%) and 11/25 (44%) patients, respectively. There was no case of central diabetes insipidus. MRI findings were seen in 47/61 (77%) patients including stalk thickening 36/61 (59%), suprasellar convexity 43/61 (70%) and heterogeneous enhancement of the pituitary gland 30/61 (49%). Other concomitant endocrine adverse events were thyroiditis (2/62, 3%), and immune-mediated diabetes (2/62, 3%).

**Table 2:** Clinical presentation, MRI findings and hormonal assessment of immune checkpoint inhibitor-related hypophysitis (*n* = 62).

Presentation	*n*/*n* tested (%)
Symptoms	
Headache	37/62 (60)
Fatigue	41/62 (66)
Visual defects	0/62 (0)
Polyuria, polydipsia	0/62 (0)
Radiographic consistent with irH	47/61 (77)
Pituitary hormone deficiencies	
TSH (central hypothyroidism)	58/62 (94)
ACTH (central adrenal insufficiency)	43/62 (69)
FSH/LH (central hypogonadism)	29/57 (50)
Prolactin	27/43 (63)
Growth hormone	11/25 (44)
ADH (diabetes insipidus)	0/62 (0)
ACTH + TSH deficiencies	39/57 (68)
ACTH + TSH + FSH/LH deficiencies	18/57 (32)
Hormonal replacement if hormone was deficient	
Steroids	43/43 (100)
Thyroid hormone	54/58 (93)
Testosterone	10/29 (34)
Initiation of high dose steroids at irH diagnosis	31/62 (50)
ICI continuation after irH diagnosis	31/62 (50%)

ICI, immune checkpoint inhibitor; IrH, immune checkpoint inhibitor-related hypophysitis.

### Predictors of irH development

**Figure 3:**
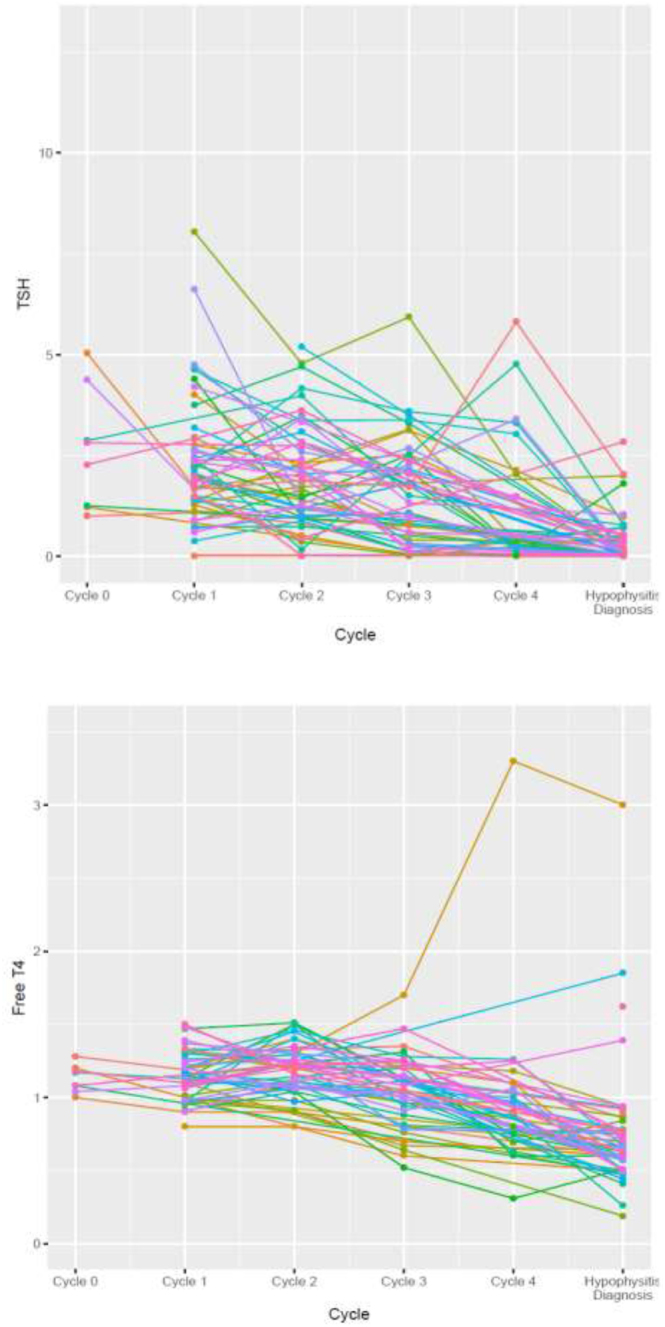
TSH and free T4 trends in hypophysitis group in correlation with cycles of immune checkpoint inhibitor administration.

Factors that could potentially be associated with irH development were analyzed. Baseline patient demographics (age, gender, and ethnicity), cancer type, and pre-existing autoimmune disease prior to ICI use were not found to be associated with irH occurrence. Therapies containing ipilimumab have a much stronger association with irH compared to those without ipilimumab (*P* < 0.01). The trend of TSH and free T4 values before each cycle of ICI administration were reviewed. For TSH, there is some evidence of an interaction between groups (irH vs without irH) and cycles of ICI administration (*P* = 0.072) although this did not reach statistical significance. For free T4, there is no evidence of an interaction between group and cycle (*P* = 0.27). In irH group, TSH decreases between cycle 1 to cycle 2 and cycle 2 to cycle 3 and was statistically significant (*P* = 0.01 and *P* < 0.01, respectively). The decrease of free T4 between cycle 1 to cycle 2 and cycle 2 to cycle 3 also achieved statistical significance (*P* = 0.07 and *P* < 0.01, respectively). The overall average decrease overtime was 0.17 units per cycle for non-irH patients, with an additional 0.21 units per cycle for irH group (Fig. 3).

### Recovery and long-term follow-up

Table 3 shows pituitary hormonal recovery patterns.

**Table 3:** Hormonal recovery in immune checkpoint inhibitor related hypophysitis group.

Hormonal axis affected (*n*)	Hormonal replacement at diagnosis		Hormonal replacement at last follow-up	Recovery based on formal testing, *n*(%)			Median time to recovery
				Yes	No	Unclear	
Thyroid (58)	Treated	54	44	10	10	34	24 weeks
	Not treated	4	0	(18.5)	(18.5)	(63)	(15.4–53.4)
				4	0	0	
				(100)	(0)	(0)	
Adrenal (43)	Treated	43	43	0	24	19	n/a
	Not treated	0	0	(0)	(56)	(44)	
				0	0	0	
				(0)	(0)	(0)	
Gonadal (29)	Treated	10	5	5	1	4	17 weeks
	Not treated	19	0	(50)	(10)	(40)	(10.7–38)
				12	2	5	
				(75)	(10)	(15)	

All 43 patients with central adrenal insufficiency received glucocorticoid therapy either physiologic or high dose at diagnosis. Thirty-five of 43 patients (81%) were tested for adrenal recovery within 1 year and 43/43 (100%) during the follow-up. Of 43 patients, 17 (39%) had adrenal recovery assessed by cosyntropin stimulation test, 10 (24%) by early morning cortisol and ACTH and 16 (37%) by random ACTH and/or cortisol. At the median follow-up time of 1 year (0.7–2.3), all patients with adrenal insufficiency remain on steroids. No case of adrenal axis recovery was observed.

Among the 58 patients with central hypothyroidism, 54/58 (93%) received thyroid hormone while 4/58 (7%) did not. All patients (58/58, 100%) had thyroid hormone testing within 1 year. At the median follow-up time of 1 year (0.6–2.3), 44/54 (81%) patients with central hypothyroidism who were started on thyroid hormone remained on treatment. Spontaneous recovery was shown in all 4 patients with central hypothyroidism who did not receive thyroid hormone at the time of diagnosis. Three patients in this group received a supraphysiologic dose of steroids upon diagnosis. In the 54 patients receiving thyroid hormone, complete recovery in thyroid function occurred in 10 (19%) of patients, no recovery in 10 (19%) and unclear recovery in 34 (63%). Median time to thyroid axis recovery was 24 weeks (15.4–53.4).

Ten of 29 (34%) patients with central hypogonadism received sex hormonal replacement while 19/29 (66%) did not. Twenty-five of 29 (86%) patients were tested for gonadal function recovery within 1 year and 25/29 (86%) during the follow-up. At the median follow-up time of 2 years (0.7–2.1), 5/10 (50%) patients with central hypogonadism who were started gonadal hormone replacement remained on treatment.

Spontaneous recovery was seen in 12/19 (63%) patients with central hypogonadism who were not started on sex hormonal replacement. In the 10 patients who received hormone therapy, gonadal function recovery was found in 5/10 (50%) of patients, no recovery 1/10 (10%) and unclear recovery 4/10 (40%). Median time to gonadal axis recovery was 17 weeks (10.7–38).

Thirty-nine of 46 (85%) patients had follow-up MRI for review in which all 39/39 (100%) showed MRI resolution with a median time of 11 weeks (7–14).

### Predictors of recovery

For this analysis, patients with unclear recovery were excluded. Age, gender, ethnicity, cancer type, ICI types and duration of use, pre-existing autoimmune diseases, low prolactin and number of hormone axis affected were not shown to be associated with hormonal recovery. At diagnosis, supraphysiologic dose cortisol was given in 31/62 (50%) patients. The use of steroids was not significantly associated with hormonal recovery (thyroid and gonadal (*P*  = 0.7 and 0.4, respectively). In 31/62 (50%) patients, ICI was continued after irH diagnosis. Discontinuation of ICI was also not associated with hormonal recovery (thyroid and gonadal (*P*  = 0.35 and 0.32, respectively) (Supplementary Table 2).

## Discussion

This study is the first to propose and validate irH diagnostic criteria, describe its clinical presentation and provide insights into recovery patterns and long-term follow-up. Hypophysitis is rare with an annual incidence of about 1 in 9 million (Hunn *et al.* 2014). Lymphocytic hypophysitis was the most common histologic variant (Caturegli *et al.* 2005). However, the emergence of irH resulted in a dramatic rise in the incidence of this disorder making it likely the most common cause of hypophysitis in the current era. With the evolving roles of ICIs in many types of cancer (Brahmer & Pardoll 2013, Azijli *et al.* 2014, Barbee *et al.* 2015), more patients are now being treated and benefiting from therapy, resulting in an increased number of patients with irH. Therefore, establishing validated diagnosis criteria followed by treatment and follow up recommendations are essential. Recently, some oncology guidelines have been developed (Puzanov *et al.* 2017, Brahmer *et al.* 2018) with an attempt to have consensus recommendations in the management of irAEs related to ICIs. However, these guidelines were based on small retrospective studies and expert opinions with no prospective data available. No endocrinology guidelines have yet incorporated irH as a new entity.

Currently, IrH diagnosis is exclusively based on a temporal relationship with ICI use and clinical information; however, there is a significant variation in its definition, severity, hormonal criteria as well as MRI findings in the published medical literature (Brilli *et al.* 2017, Sznol *et al.* 2017, Alessandrino *et al.* 2018, Kanie *et al.* 2018). Due to inconsistency in irH definition, it is challenging to lay a solid background for recommendations of treatment and long-term follow-up. Ideally, a prospective study is needed; however, recruiting enough patients to conduct such study is challenging and time-consuming. The present study is the largest cohort study with long-term follow-up on irH. As a single institution study, this provides unique opportunities to have consistent laboratory data, centralized MRI interpretation and endocrine management, which was lacking in the majority of previously published studies. The patients evaluated for irH are cancer patients who may have non-specific symptoms such as fatigue, weight loss, anorexia, etc. and may be on therapies that can affect hormonal work up such as GnRH agonists and glucocorticosteroids. In the studied population, we sought to use strict diagnostic criteria to identify true irH cases. Diagnosis based on symptoms only or a single laboratory abnormality or MRI change might lead to high sensitivity, but low specificity. Symptoms such as headache or fatigue, although often seen in irH patients as reported, are non-specific and can be seen quite common in cancer patients. The proposed criteria are, therefore, strict and require multiple elements including symptoms, MRI findings and hormonal work up to increase the specificity of the diagnosis. When applied to our study population, the criteria have shown excellent performance by accurately identifying 61/62 (98.3%) cases with irH at initial evaluation. The one patient who met criteria later on but not at initial evaluation might reflect a well-reported finding that sometimes pituitary or brain MRI changes may precede clinical or hormonal changes (Faje *et al.* 2014). It is important that clinicians continue to have close follow-up in patients suspected for irH but might not have full manifestation at the first evaluation. This includes patients who had no symptoms or presented with only one hormone insufficiency at the initial assessment. We recognize that there is a group of patients, although did not meet the criteria, had confirmed isolated pituitary hormone insufficiency. IrH could not be excluded in those patients or perhaps one axis can be impacted without affecting others. In these cases, irH might have been mild and not progress further. When including these cases, the proposed criteria still identify the majority of irH cases (61/69, 88%).

In our study, irH diagnosis would have been missed in 20/61 (33%) patients if no MRI were obtained. This highlights the importance of obtaining both a hormonal study and a pituitary MRI if irH is suspected. We strongly recommend obtaining pituitary MRI in all cases suspected of irH. This is in contrast with the recent ASCO/NCCN guideline on the management of immunotherapy-related toxicities in which MRI consideration is in selected groups of patients with multiple endocrine abnormalities with or without new severe headaches or complaints of vision change (Brahmer *et al.* 2018). Importantly, radiographic changes can precede clinical presentation or hormonal dysfunction. The prevalence of irH was not assessed in this study. However, it is noted that the anti CTLA-4 mAbs (ipilimumab and tremelimumab) as monotherapy or in combination with anti PD-1 mAbs (ipilimumab plus nivolumb) represent the majority of the study population with only few cases of single-agent anti PD-1 mAb being reported. Therapies containing ipilimumab have a much stronger association with IrH compared to those without. This is consistent with the published data on the higher prevalence of irH that occurs with anti CTLA-4 mAbs (Iwama *et al.* 2014). A possible explanation is that normal pituitary tissues express ectopic CTLA-4 protein and binding of ipilimumab (anti CTLA-4 IgG1) to native CTLA-4 proteins on normal pituitary tissue lead to the activation of the classic complement pathway resulting in inflammation (Iwama *et al.* 2014, Caturegli *et al.* 2016, Byun *et al.* 2017).

Demographics, clinical presentation, hormonal profiles and MRI findings in the studied population are largely similar to what have been reported in the literature (Faje *et al.* 2014, Albarel *et al.* 2015, Min *et al.* 2015). The majority of patients being men is likely a reflection of the male predominance in common types of cancer in which ICIs were used (melanoma and prostate cancer). Thyroid axis seems to be most commonly affected (58/62, 94%), followed by central adrenal insufficiency (43/62, 69%) and hypogonadism (29/57, 50%). Diabetes insipidus was not present in our study. To date, there have been only two cases of central diabetes insipidus reported in patients on avelumab (an anti-PD-L1 mAb) (Zhao *et al.* 2018) and ipilimumab (Dillard *et al.* 2010). MRI is a useful diagnostic tool, especially when baseline or follow-up MRIs are available for comparison. Features associated with irH are gland height change, suprasellar bulge, stalk thickening, heterogenous enhancement and parasellar extension. This is also the first study to report these specific MRI findings in a large cohort.

In clinical practice, TSH and free T4 are frequently checked prior to each cycle of ICI administration. Our study shows that in TSH values between groups (irH vs without irH) and cycles of ICI, there is evidence of an interaction although this did not reach statistical significance. In the irH group, the decrease of TSH and free T4 between cycle 1 to cycle 2 and cycle 2 to cycle 3 was statistically significant. This suggests that TSH and free T4 trend can be used to assist in the early diagnosis of irH. In our practice, a TSH drop between cycles 1 and 3, although they can still remain within normal range, is frequently observed a few weeks before patients develop symptoms or other hormone deficiency. These patients need to be followed closely and perhaps a full pituitary panel could be checked for early detection of irH. Evaluation for hormonal recovery can be very challenging as no standardized procedure for recovery testing exists to date and many patients are not tested. Hormonal recovery described in prior studies is solely based on the continuation of hormonal treatment at the time of follow-up, which could clearly miss cases of recovery in the absence of formal testing (Faje *et al.* 2014, Lam *et al.* 2015, Brilli *et al.* 2017). In the current study, in addition to evaluating recovery based on the continuation of treatment at the time of last follow-up, we propose criteria for the recovery of various hormonal deficiencies. Our patients were frequently tested for hormonal testing as presented above. However, the process of hormone re-assessment was not quite uniform due to the study’s retrospective nature. No significant changes in recovery rates were noted based on our recovery criteria as compared with prior criteria of continuation of hormonal treatment at the time of last follow-up. Interpretation of recovery remains challenging due to the lack of uniform testing.

In this cohort, no cases of recovery in the adrenal axis were found. Complete thyroid axis recovery was achieved in a subset of patients (24%) with recovery time ranging from 15 weeks to 53 weeks. Recovery in the gonadal axis took place in 71% of patients who were tested. Median time to recovery was 17 weeks (10.7–38). In other studies, hormone recovery has been documented with the incidence varying from 5% to 100% for the thyroid axis (Faje *et al.* 2014, Brilli *et al.* 2017), 0% to 27% for the adrenal axis (Weber *et al.* 2013, Albarel *et al.* 2015) and 13 % to 60 % for central hypogonadism (Faje *et al.* 2014, Brilli *et al.* 2017). However, it is important to know that no clear definition for hormone recovery was used in any of these studies. Since the majority of cases with thyroid and gonadal hormonal recovery took place within the 1st year and as early as 3 months, re-assessment for hormonal recovery every 3 months in the 1st year and every 6 months thereafter is recommended. Indeed, in our practice, TSH rise during the follow-up could be suggestive of potential thyroid axis recovery. Thyroid hormone replacement should be reassessed for a dose reduction or drug discontinuation with repeat testing in 6 to 8 weeks. Although adrenal recovery was not observed in this study, almost 50% of patients had only random cortisol and ACTH testing limiting a true assessment. Few cases of adrenal axis recovery were reported in the literature (Ryder *et al.* 2014). Based on these data, adrenal insufficiency appears to be a lifelong sequela of irH. Until more data are available, we recommend standard testing with either cosyntropin stimulation test or early morning ACTH and cortisol with an appropriate holding of corticosteroid dose every 3 months in the 1st year and every 6 months in the 2nd year to identify those patients who may have a recovery and could potentially stop steroid therapy. The fact that many patients with central hypogonadism recovered might be explained by the recovery of their acute illnesses which might have been the main cause of their suppressed sex hormones or a higher chance of recovery from gonadal cells in irH. Gonadal hormone replacement might not be immediately needed in irH-related central hypogonadism as many patients can potentially recover without sex hormone replacement. Sex hormone function and treatment can be reassessed in 2–3 months after irH diagnosis.

None of the baseline characteristics or clinical and MRI features of irH at the time of diagnosis were found to be associated with the prediction of hormonal recovery. Investigation for better predicting tools is warranted. A study has demonstrated the role of autoantibodies recognizing TSH, FSH and ACTH-secreting cells as one possible mechanism of irH (Iwama *et al.* 2014). In this study, among 20 patients with negative pituitary antibodies at baseline, 7/7 (100%) patients with clinical irH developed antibodies at the time of irH diagnosis. Pituitary antibodies remained negative in the remaining 13 patients without clinical irH. It may be helpful to study these antibody titers over time in correlation with irH diagnosis, progression and recovery.

In our study population, high dose steroids and discontinuation of ICIs were not shown to be significantly associated with hormonal recovery. Similar finding was also reported in a smaller size retrospective study (Min *et al.* 2015). A recent study in 2018 reported a negative impact of high-dose steroids on overall survival and time to treatment failure in a cohort of melanoma patients with ipilimumab-induced hypophysitis (Faje *et al.* 2018). This suggests that high dose steroids may not be necessary in all cases, especially for patients without severe symptoms. In those with severe or life-threatening symptoms such as visual changes, severe headache or adrenal crisis, hospitalization, high-dose steroids administration may be warranted.

It is unknown if treatment with glucocorticoids for anti-inflammatory effects in the very early phase of IrH in which radiographic changes in brain/pituitary MRI are the only manifestation that could theoretically decrease acute inflammation in the pituitary gland with potential sparing hormonal deficiencies. Patients with a diagnosis of irH can continue their ICI treatment after appropriate workup and treatment have been initiated. This is in line with recommendations in the guideline for ICI toxicity management from the Society of Immunotherapy of Cancer (Puzanov *et al.* 2017).

**Figure 4:**
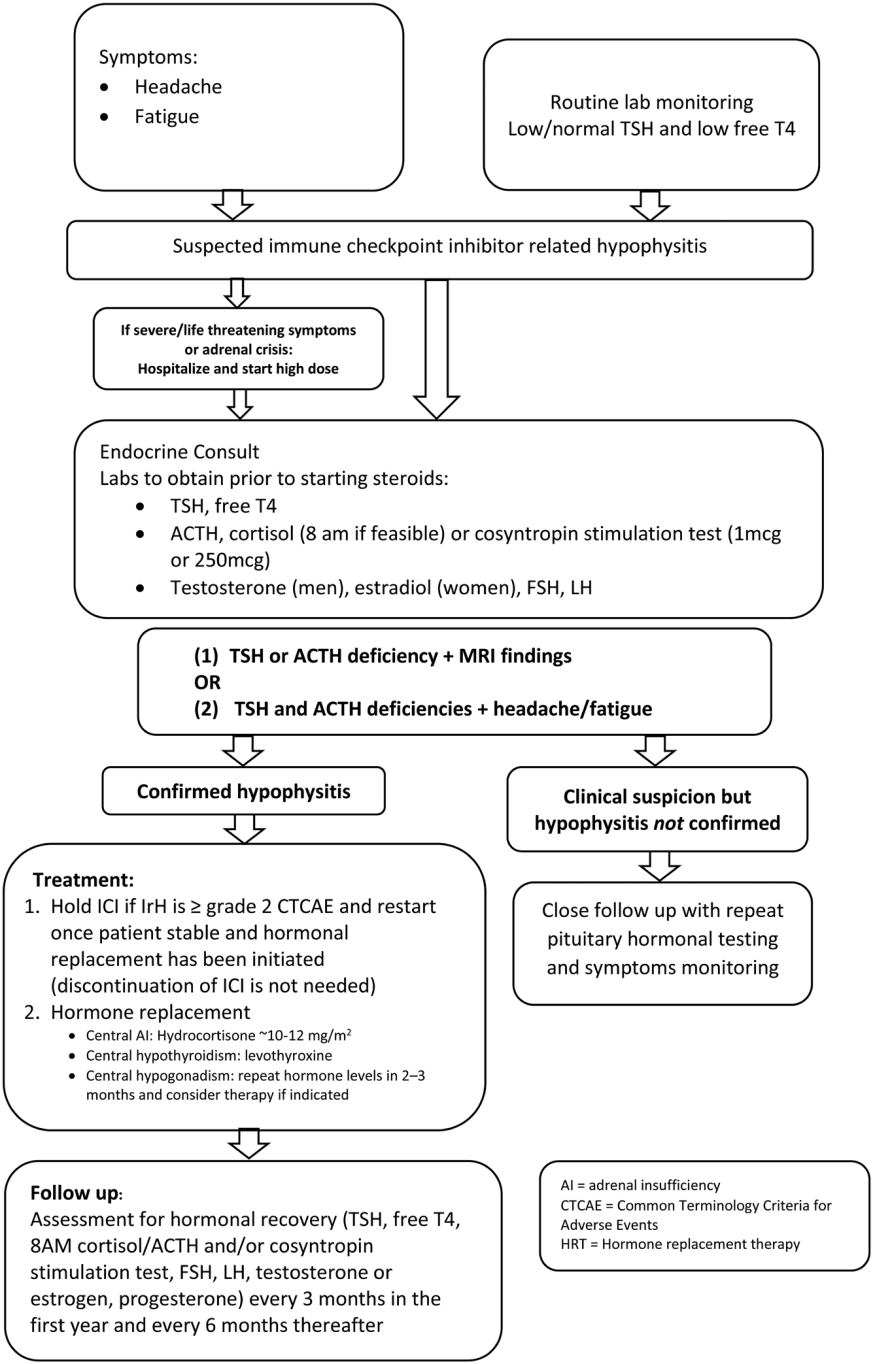
Diagnosis and management of immune-related hypophysitis.

Based on our current data as well as previous reported data, a proposed guideline for diagnosis, treatment and follow-up is shown in Fig. 4.

We recognize certain limitations of this study. Given its retrospective nature, there might have been potential biases such as inconsistency in chart documentation, variations in the management of irH, missing data, loss of follow-up, and inconsistency in hormonal recovery evaluation. Currently, irH is a clinical diagnosis with no confirmatory histology in patients or an alternative method to confirm at cellular levels. However, a pituitary biopsy is neither necessary nor indicated in irH given the risks outweighing the benefits. Our study has shown that the proposed criteria have a great correlation with what is seen in clinical practice including patient’s symptoms, hormonal and MRI findings at presentation and their progression over time with long-term data.

## Conclusions

IrH remains a diagnostic challenge. The diagnosis requires a full assessment of the patient’s symptoms, pituitary hormonal work up and MRI changes. The proposed criteria have shown excellent performance and can be used at initial evaluation of suspected irH cases: (1) ACTH or TSH deficiency plus pituitary MRI findings consistent with irH or (2) both ACTH and TSH deficiencies plus symptoms of headache or fatigue in the absence of MRI findings/evaluation. However, patients with high clinical suspicion for irH who may not meet the criteria at initial evaluation should be followed closely. High-dose steroids may not always be needed in cases without severe or life-threatening symptoms. Hormonal recovery can be achieved in a subset of patients, therefore, periodic assessment and long-term follow-up are recommended. Development for better predicting tools for irH occurrence and recovery is warranted.

## Supplementary Material

Supplemental TABLE 1- Reference rages at the University of Texas MD Anderson Cancer Center labs

Supplemental TABLE 2a – analysis of potential factors affecting thyroid hormone recovery (n=24)

Supplemental Table 2b- analysis of potential factors affecting gonadal hormone recovery (n=20)

## Declaration of interest

A D received research funding from Idera Pharmaceuticals, Nektar Therapeuticsm, Bristol-Myeres and Squib, Apexigen and Pfizer. S K S: consulting/advisory role for Apricity Health, Compugen, Dendreon, Janssen, Baylor and Polaris. Other (joint scientific committee): Janssen, Polaris. The other authors have nothing to disclose.

## Funding

Supported by the National Institutes of Health http://dx.doi.org/10.13039/100000002/National Cancer Institute http://dx.doi.org/10.13039/100000054 under award number P30CA016672.

## Ethics approval and consent to participate

This study was approved by the Institutional Review Board of the University of Texas MD Anderson Cancer Center.

## Consent for publication

Not applicable. This manuscript does not contain individual details, images or identification.

## Availability of data and material

The datasets during and/or analyzed during the current study available from the corresponding author on reasonable request.

## Author contribution statement

H N and R D collected, analyzed the data and were major contributors in writing the manuscript. K S, G L S, and D K reviewed and analyzed data on brain/pituitary MRI. S G W reviewed and analyzed the data. M I H, M E C, M A H, N L B, P I, S K S, A D contributed in reviewing the data and editing the manuscript. M J P collected data. R B and S Z performed data analysis. All authors read and approved the final manuscript.
