# Supplementary material for: Immune checkpoint inhibitor related hypophysitis: diagnostic criteria and recovery patterns
Source: Endocr Relat Cancer. 2021 Apr 23;28(7):419–31. doi: 10.1530/ERC-20-0513 (PMC8183642; doi:10.1530/ERC-20-0513)
Supplement: Supplemental TABLE 1- Reference rages at the University of Texas MD Anderson Cancer Center labs [file supplementary_table_1.pdf]

Supplemental TABLE 1- Reference rages at the University of Texas MD Anderson Cancer Center labs

Blood test	Reference ranges at MDACC labs
ACTH	0-46 pg/mL
Cortisol morning (8am)	4.3 - 22.4 mcg/dL
TSH	0.27 - 4.20 mcunit/mL
T4 Free	0.93 - 1.70 ng/dL
Testosterone	Male (age 20-49): 249-836(ng/dl) , (age ≥ 50): 193-740<br>Female (age 20-49): 8-48, (age ≥ 50): 3-41
FSH	Female: Follicular 3.5-12.5 (mIU/mL), ovulation 4.7-21.5, luteal: 1.7-7.7, post menopause: 25.8-134.8
LH	Female: Follicular 2.4-12.6 (mIU/mL), ovulation 14-95.6, luteal: 1-11.4, post menopause: 7.7-58.5
Prolactin	(nonpregnant female): 4-15.2 ng/mL
Insulin-Like Growth Factor 1-Mayo:	32 - 200 ng/mL
