# Supplementary material for: Immune checkpoint inhibitor related hypophysitis: diagnostic criteria and recovery patterns
Source: Endocr Relat Cancer. 2021 Apr 23;28(7):419–31. doi: 10.1530/ERC-20-0513 (PMC8183642; doi:10.1530/ERC-20-0513)
Supplement: Supplemental TABLE 2a – analysis of potential factors affecting thyroid hormone recovery (n=24) [file supplementary_table_2a.pdf]

Supplemental TABLE 2a – analysis of potential factors affecting thyroid hormone recovery (n=24)

	**Number**	**Recovery**	**Hazard ratio**	**Lower confidence interval (CI)**	**Upper CI**	**P value**
Age	24	14	0.975	0.927	1.026	0.3
Sex						
• Female	4	3	--	--	--	--
• Male	20	11	0.785	0.215	2.86	0.7
Race						
• Caucasian	22	12				
• African American	1	1	7.682	0.789	74.793	0.07
• Hispanic	1	1	2.5	0.318	21.045	0.3
Cancer type						
• Melanoma	15	10	--	--	--	--
• Renal cell	1	0	0	0.002	2.399	0.3
• Prostate cancer	6	3	0.5	0.148	1.784	0.3
• Other	2	1	0.1	0.001	1.428	0.1
ICI						
• Any Ipilimumab	21	12	--	--	--	--
• No Ipilimumab	3	2	1.8	0.383	8.582	0.4
Pre-existing autoimmune disease						
• No	22	14	--	--	--	--
• Yes	2	0	4.5	0.837	18.19	0.07
Low prolactin						
• No	7	5	--	--	--	--
• Yes	10	4	0.3	0.077	1.405	0.1
High dose steroids						
• No	8	5	--	--	--	--
• Yes	16	9	0.818	0.27	2.47	0.7
Number of hormone axis affected	24	14	0.679	0.308	1.5	0.3
