# Supplementary material for: Immune checkpoint inhibitor related hypophysitis: diagnostic criteria and recovery patterns
Source: Endocr Relat Cancer. 2021 Apr 23;28(7):419–31. doi: 10.1530/ERC-20-0513 (PMC8183642; doi:10.1530/ERC-20-0513)
Supplement: Supplemental Table 2b- analysis of potential factors affecting gonadal hormone recovery (n=20) [file supplementary_table_2b.pdf]

Supplemental Table 2b- analysis of potential factors affecting gonadal hormone recovery (n=20)

	Number	Recovery	Hazard ratio	Lower confidence interval (CI)	Upper CI	P value
Age	20	17	1.006	0.953	1.062	0.8
Sex<br>• Female<br>• Male	 1<br>19	 1<br>16	 --<br>0.249	 --<br>0.028	 --<br>2.233	 --<br>0.2
Race<br>• Caucasian<br>• African American	 19<br>1	 16<br>1	 <br>4.014	 <br>0.448	 <br>35.98	 <br>0.2
Cancer type<br>• Melanoma<br>• Renal cell<br>• Other	 17<br>2<br>1	 15<br>2<br>0	 --<br>0.06<br>0.205	 --<br>0<br>0.002	 --<br>0.78<br>1.57	 --<br>0.02<br>0.1
ICI<br>• Any Ipilimumab<br>• No Ipilimumab	 16<br>4	 14<br>3	 --<br>0.977	 --<br>0.274	 --<br>3.49	 --<br>0.9
Pre-existing autoimmune disease<br>• No<br>• Yes	 18<br>2	 16<br>1	 --<br>1.583	 --<br>0.189	 --<br>13.25	 --<br>0.6
Low prolactin<br>• No<br>• Yes	 6<br>10	 6<br>8	 --<br>0.706	 --<br>0.235	 --<br>2.12	 --<br>0.5
High dose steroids<br>• No<br>• Yes	 11<br>9	 10<br>7	 --<br>0.615	 --<br>0.209	 --<br>1.81	 --<br>0.3
Number of hormone axis affected	20	17	0.452	0.157	1.296	0.1
